# Effects of a multi-level intervention on the pattern of physical activity among in-school adolescents in Oyo state Nigeria: a cluster randomised trial

**DOI:** 10.1186/s12889-017-4781-y

**Published:** 2017-10-23

**Authors:** Mojisola Morenike Oluwasanu, Oladimeji Oladepo

**Affiliations:** African Regional Health Education Centre, Department of Health Promotion and Education, Faculty of Public Health, College of Medicine, University of Ibadan, Ibadan, Nigeria

**Keywords:** Self-reported physical activity, Objectively measured physical activity, Physical fitness, In-school adolescents, Multi-level intervention

## Abstract

**Background:**

Physical inactivity contributes to the global burden of non-communicable diseases. The pattern of physical activity in adulthood are often established during adolescence and sedentary behaviours in the early years could influence the development of diseases later in life. Studies on physical activity in Nigeria have focused largely on individual behaviours and the effects of school-based interventions have not been well investigated. The aim of the proposed study is to identify factors influencing; and evaluate the effects of a multi-level intervention on the physical activity behaviours of in-school adolescents in Oyo state, Nigeria.

**Methods:**

The study will adopt a cluster randomised controlled trial design and schools will serve as the unit of randomisation. The sample size is 1000 in-school adolescents aged 10–19 years. The study will be guided by the socio-ecological model and theory of reasoned action and baseline data will be obtained through a mixed methods approach comprising a cross sectional survey to document the self-reported physical activity levels coupled with objectively measured physical activity levels using pedometers for a subset of the sample. Other measurements including weight, height, waist and hip circumferences, fitness level using the 20-m shuttle run test (20-mSRT) and blood pressure will be obtained. The schools’ built environment and policy support for physical activity will be assessed using structured questionnaires coupled with key informant interviews and focus group discussions with the school authorities. Baseline findings will guide the design and implementation of a 12-week multi-level intervention. The primary outcome measures are self–reported and 7-day objectively measured physical activity. Other secondary outcome measures are body-mass-index for age, waist-to-hip ratio, cardioresiratory fitness and blood pressure. The association between behavioural factors and physical activity levels will be assessed. Follow-up measurements will be taken immediately after the intervention and 3-months post intervention.

**Discussion:**

Physical activity behaviours of adolescents in Nigeria are influenced by multiple factors. There is an urgent need for effective school-based interventions with a potential to improve the physical activity behaviours of adolescents in Nigeria and other low and middle income countries.

**Trial registration:**

Pan African Clinical Trial Registry. Trial registration number: PACTR201706002224335, registered 26 June 2017.

## Background

Nigeria, a developing country with a population of 170 million and a third of its populace in the 10–24 years age group contributes significantly to the global burden of non-communicable diseases (NCDs) [1, 2]. According to the Global Status Report on Non-communicable diseases, Nigeria had an estimated 792,600 NCDs related deaths in 2008 and this high mortality figure is linked to four modifiable risk factors namely *physical inactivity, unhealthy diets, tobacco and harmful alcohol use* [2]. These risk factors are typically initiated during adolescence and set the stage for unhealthy behaviours which persist into adulthood and diseases later in life [3].

According to the National Strategic Framework on the Health and Development of Adolescents and Young People in Nigeria, there is a gradual increase in the number of physically inactive, overweight, young children and adolescents in Nigeria [4]. Obesity in childhood and adolescence has been linked to cardiovascular diseases, type-2 diabetes, cancer and depression [5–8]. Findings from recent studies conducted among adolescents in different Nigerian states lend credence to the growing epidemic; the reported prevalence of overweight and obesity among adolescents in Benue state was 9.7% and 1.8% [9]; 13.8% and 9.4% in Lagos state [10] and 5.8% and 1.1% in Ondo state [11] respectively.

Compared to other developed regions of the world, the reported prevalence of obesity among adolescents in Nigeria is low however; this is rapidly increasing due to the corresponding rise in the behavioural antecedent risk factors specifically *unhealthy diets and physical inactivity*. A study by Odunaiya et al., (2010) among in-school adolescents in Oyo State, Nigeria found that, 38%, 58.8% and 3.2% engaged in low, moderate and high intensity physical activity behaviours respectively [12]. Furthermore, 8.8% were overweight and 1.2% were obese and body mass index was inversely associated with physical activity levels. Adeniyi et al. (2011) also found a significant level of physical inactivity among students in Ibadan, Oyo State and this was linked to individual and school factors [13]. According to Adeniyi et al, the physical activity levels of the participants varied; 53.8%, 38.8% and 7.4% engaged in low, moderate and high intensity physical activity behaviours respectively [13]. Physical inactivity was higher among students in private schools and associated with depression. The increasing level of physical inactivity in Nigeria and many other countries in the world is largely due to motorisation, urbanisation, automation of daily activities and greater opportunities for sedentary behaviours and underscores the need to address this growing burden [14–16].

In 2013, the World Health Assembly endorsed a resolution calling upon member states to address the needs of the youth by prioritising the surveillance of trends and determinants of non-communicable diseases and adopting a lifecourse approach in its prevention and control [17]. Evidence points to adolescence as a crucial period in the development of NCDs and underscores the need to institute or intensify surveillance of risk factors and implement population wide interventions to reduce the morbidity and mortality associated with these diseases. Schools have been identified as a setting for influencing the physical activity behaviours of young people but several factors influences its role in this regard. Numerous recommendations have been proposed to address physical inactivity and unhealthy diets within the school setting however; most of these strategies have targeted the individual level resulting in relatively modest changes in behaviours.

There is a global call to address the root cause of unhealhy diets and physical inactivity by implementing population wide interventions within health promoting setting such as schools and workplaces and this requires profound policy changes and leadership support [15]. In order to reduce the impact of the major risk factors for NCDs, the World Health Assembly adopted the Global Strategy on Diet, Physical Activity and Health and the School Policy Framework on Diet and Physical Activity [18]. Paragraph 49 of this document states that*: “School policies and programmes should support the adoption of healthy diets and physical activity. Schools are encouraged to provide students with daily physical education and should be equipped with appropriate facilities and equipment. Governments are encouraged to adopt policies that support healthy diets at school and limit the availability of products high in salt, sugar and fats”*. The extent of implementation of this initiative in Nigeria is currently low.

A systematic review of few studies conducted in high income countries found strong evidence that multi-component interventions and policies were effective in promoting physical activity among students [19]. However; this intervention has not been tested in Nigeria. A study conducted by Gillis et al. (2013) among international physical activity experts using a twin-panel Delphi methodology ranked *policy and/or environmental change and their influence on children’s physical activity and sedentary behaviours* second on the list of 29 international research priorities on child and adolescent physical activity behaviours [20]. This underscores the justification for this study. Research studies on the physical activity behaviours of young people in Nigeria have focused solely on individual behaviours [12, 21] without analyzing critically, the influence of social-cultural, institutional, environmental and policy level factors resulting in an unbalanced attention to individualised behaviour change strategies instead of a true public health intervention which requires systems approach [22]. These underscore the importance of this study which is designed to identify the socio-ecological factors (*individual, social, built environment and policy*) influencing the physical activity behaviours of in-school adolescents and to evaluate the effect of a multi-level intervention on the physical activity and fitness levels of in-school adolescents in Oyo state, south western Nigeria.

## Methods

### Study research design

The proposed study will utilise a cluster randomised trial design to test the effect of a 12-week multi-level intervention on the physical activity and fitness levels of in-school adolescents aged 10–19 years in twenty two public and private-owned secondary schools in Oyo state, south western Nigeria. Secondary schools will be the unit of randomisation, and these will be assigned to the multi-level intervention or control arm (no intervention) with longitudinal data collection for index or cohort children randomly selected from the student population. Prior to the experimental study, preliminary data will be collected on the physical activity behaviours of in-school adolescents and the socio-ecological factors influencing the school physical activity environment. This will guide the design of a multi-level, multi-component intervention which will run for 12 weeks with assessments conducted at baseline, post-intervention and at 3-months follow-up. The design, conduct and reporting of this study will adhere to the Consolidated Standards of Reporting Trials (CONSORT) guidelines. Ethical approval was obtained from the Oyo State Research Ethical Review Committee (AD13/479/890) and permission from the Oyo State Ministry of Education. The trial is registered with the Pan African Clinical Trial Registry.

### Study site

**Fig. 1:**
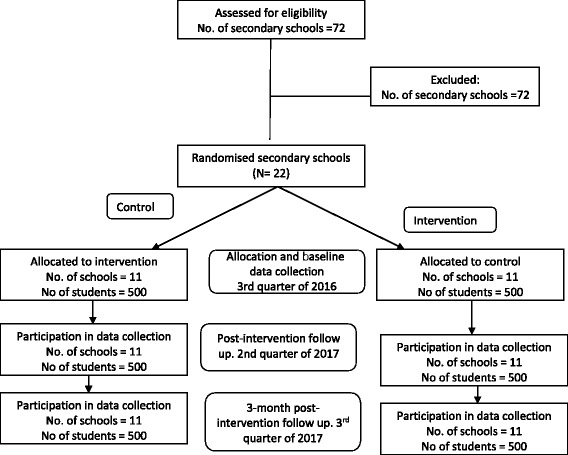
CONSORT flowchart describing progress of participants through the study

The study sites are Ibadan and Ogbomosho, urban cities located in Oyo State, the South-West geopolitical region of Nigeria. It is one of the three States carved out of the former Western region of Nigeria in 1976. Oyo State consists of 33 Local Government Areas. The State covers a total of 27,249 km^2^ of land mass and has a population of about 4.5million. The people of Oyo State may be divided into five broad groups which are: Ibadan, Ibarapa, Oyo, Oke-Ogun and Ogbomoso. The state has four universities, (one federal government university, a state government university and two private universities); four polytechnics (one state government owned and three private polytechnics) and over 500 public and private secondary schools (Fig. 1).

### Study population

The study population are in-school adolescents (10–19 years) in co-educational public and private schools. In addition, officials of the Ministry of Education and School Principals will be interviewed using a key informant interview guide while Physical and Health Education Teachers will complete semi-structured questionnaires. Focus group discussions will be held with Classroom Teachers.

#### Inclusion Criteria

- In-school adolescents in public and private schools

#### Exclusion Criteria

- Out-of-school youths
- Adolecents in schools for the disabled

### Sample size for the intervention study

The minimum sample size for this study was obtained using the formula for calculating sample size for two proportions with the aim of detecting a difference in outcomes between the groups [23]. The effect size of a similar study which was conducted in Denmark [24] and the prevalence of moderate and high intensity physical activity reported by Odunaiya et al., 2010 [12] were used for calculating the minimum sample size. A multi-level intervention to promote physical activity among in-school adolescents has not been conducted in Nigeria and the effect size is unknown. This justifies the use of the value reported in Denmark [24]. A *multilevel intervention is defined as an intervention* that addresses at least three levels of the multilayer system. It target at least three sources of influence that may ultimately result in improved health behaviour and outcomes [25].

Intracluster correlation coefficient (ICC) is a measurement that captures between-cluster and within-cluster variability in outcome and is required for sample size calculation in clustered studies that comprise exposed or unexposed clusters [26–29]. To account for this, the intracluster class correlation reported by Oyeyemi et al., (2014) which assessed participants’ self-reported active transportation to school and leisure-time Moderate-to-Vigorous Physical Activity (MVPA) among in-school adolescents was used to estimate the within and between school variation in physical activity in order to control for clustering within the schools (ICC = 0.38) [30]. The formula is outlined below:

$$ \mathrm{n}=\frac{{\left({Z}_{\alpha /2+}{Z}_{\beta}\right)}^2\left[{\mathrm{P}}_1\left(\ 1-{\mathrm{P}}_1\right)+{\mathrm{P}}_2\left(\ 1-{\mathrm{P}}_2\right)\right]}{{\left({\mathrm{P}}_{1\hbox{--} }{\mathrm{P}}_2\right)}^2}\times \left[1+\left({\mathrm{m}}_{\mathrm{o}}-1\right)\times \mathrm{ICC}\right]. $$

where Z_α/2_ is the critical value of the normal distribution at α/2 (e.g. for a confidence level of 95%, α is 0.05 and the critical value is 1.96), Z_β_ is the critical value of the normal distribution at β (e.g. for a power of 80%, β is 0.2 and the critical value is 0.84) and P_1_ and P_2_ are the expected sample proportions of the two groups.

Z_α/2_ = 1.96 at 5% level of error.

Z_β_ = 0.84

P_1_ = 62% i.e. the proportion of the participants in the unexposed (control) group who are either moderately or vigorously physically active *(58.8% engaged in moderate physical activity levels while 3.2% engaged in high physical activity levels based on the study by Odunaiya, et al., 2010)* [12].

P_2_ = 72% i.e. the proportion of the participants in the exposed (experimental) group who are expected to become physically active 12 weeks after the intervention. This translates to the effect size i.e. 10% increase in the outcome measure of physical activity at follow up [24].

*ρ* = 0.38 [30].

The design effect or variance inflator factor = [1+ (k-1) x*ρ*

k = approximately the average number of individuals to be sampled per cluster. This will be 50 per school.

Three hundred and thirty six per group is the minimum sample size needed to detect whether the stated difference exists between the two proportions. A total of one thousand in-school adolescents will be selected for the study (500 for the control and 500 for the experimental group) to account for loss to attrition and increase the precision for the point estimate for physical activity behaviours among the adolescents.

### Blinding and sampling technique

Multistage sampling technique will be used for the study. Nigeria is divided into six regions with approximately six states each. The south west region was purposively selected for this study.

Step 1: The study state was selected randomly from the six states in the south west region of Nigeria.
Step 2: Two comparable Local Government Areas (LGAs) in the project state will be selected using simple balloting. The list of LGAs was stratified based on their socio-economic status and level of urbanisation. One of the LGAs will be the experimental while the other will serve as the control.
Step 3: The list of schools will be obtained for the selected LGAs and stratified by ownership i.e. public and private and population size. Twenty two schools will be pair-matched based on their ownership (i.e. public and private) and population size and randomly allocated to either the experimental (11 schools) or control (11 schools) group by simple randomisation by an independent researcher not involved in the study.
Step 4: The male and female student ratio for the selected schools will be obtained and used for the determination of students to be selected for each school disaggregated by sex. The selected in-school adolescents will serve as the index children though all the students in the schools allocated to the experimental group will be exposed to the intervention.
Step 5: The number of arms for classes from each school will be documented and two arms of classes at each level in the school will be selected by balloting for the study.
Step 6: Study participants in each class will be selected using systematic random sampling technique based on the male to female student ratio and expected cluster size for the school.
Step 7: The selected respondents will be enrolled as a cohort in the experimental and control schools and followed up after the 12-week intervention.

### Data collection

The data collection will be at three time points the first will be at baseline (T0), the second will be immediately after the 12-week intervention (T1) while the third will be three months after the intervention (T2). Details in Table 1. The data collection will employ a mixed methods approach including:

(i) A desk review of relevant state and national NCDs prevention policies and guidelines on physical activity and an assessment of the level of coherence of the School Health Promotion policy with these policies. Other documents which will be reviewed include the Physical Health Education curriculum and school timetable to assess the opportunities for PA during school hours. The policies which regulate the construction of schools will also be reviewed to assess the extent to which these documents regulates or influences the built environment and physical activity levels in school.
(ii) Physical and Health Education Teachers will complete the School Physical Activity Policy Assessment tool (S-PAPA) – a standardised semi-structured tool which assesses physical education, recess and other physical activity opportunities before, during and after school [31]. For this study, only two components will be assessed - physical education and recess. Scores on the S-PAPA tool will be tabulated based on policies and practices identified as being related to children’s physical activity during school [32, 33]. Total point scores will also be either median-split into high- and low or stratified by percentile for analysis [34].
(iii) Focus Group Discussions and In-depth interviews will be held with teachers and school principals respectively to assess their opinion about social norms and support and the extent of implementation of physical activity policies and programmes in schools, level of funding, and the frequency of engagement in structured physical activity. Observations will be conducted to document facilities for physical activity in the schools using the Sport, Physical Activity and Eating behaviour: Environmental Determinants in Young people (SPEEDY) checklist. The SPEEDY checklist measures the built environment of schools and can be scored to quantify environmental support for physical activity [35]. The checklist assesses school-level environmental variables such as *Walking Provision, Cycling Provision, Aesthetics, Sport and facility provision, Other facility and Design of the school grounds* [36] The scores from each of the key variables will be dichotomised into low and high based on percentiles, and the school physical activity suitability index will be computed [36, 37].
(iv) A cross sectional survey will be conducted among the students using an adapted version of the *Physical Activity Questionnaire for Adolescents (PAQ-A)* [38] to generate self-reported data on the level of physical activity among young people. The PAQ-A is a self-administered, 7-day recall instrument. It was developed to assess general levels of physical activity for high school students approximately 13 to 19 years of age. It assesses the frequency of participation in physical activity during spare time, physical education period, lunchtime, after school, in the evenings and on weekends. A summary of the physical activity score is generated from the mean of 8 items, and ranges from 1 to 5, with higher scores indicating more frequent participation in physical activity [38]). The physical activity levels will be categorised into three; low physical activity level will be those who score between 1 to 1.9 while moderate and high physical activity levels will be those who score between 2 to 3.9 and 4 to 5 respectively [13]. For some statistical analyses, the self-reported physical activity level will be treated as a continuous variable. In a study to establish the convergent validity of the PAQ-A, the instrument was found to be significantly correlated to all self-report measures (including activity rating, *r* = 0.73; Leisure Time Exercise Questionnaire, *r* = 0.57; and 7-day physical activity recall interview, *r* = 0.59) [39]. The survey will also assess the frequency of engagement in structured physical activity from two time points (baseline and follow up) as the primary outcome measure. Based on the theory of Planned Behaviours which is the theoretical framework guiding this intervention, other variables which will be incorporated into the Physical Activity Questionnaire for Adolescents (PAQ-A) are the measures for attitude, subjective norms, perceived behavioural control and self-efficacy. A research tool which was theoretically derived by Motl et al., 2000 based on Theory of Reasoned Action (TRA) and Planned Behaviour (TPB) and Social-Cognitive Theory (SCT) will be used to assess the social-cognitive factors among this young people. This questionnaire has been tested and evidence provided for its factorial validity as a unidimensional measure of attitudes, subjective norms, perceived behavioural control and self-efficacy about physical activity among adolescent girls [40]. The core variables i.e. *knowledge of health benefits of physical activity, attitude, subjective norms, perceived behavioural control and self-efficacy about physical activity* will be analysed by scoring and summing up all question items and cut-offs will be determined using percentiles.
  The survey will be complemented with focus group discussions to assess their opinions about the individual, social, community, institutional and policy factors which influences the physical activity behaviours of young people.
(v) Anthropometric and other physical measurements (body-mass-index for age, waist-to-hip ratio, blood pressure) will be documented. Anthropometric measurements will be taken twice and averaged; for discrepancy >10%, a third measurement will be taken. The height and weight will be assessed using standard anthropometric procedures. Height will be measured using a portable height meter/stadiometer. Students will be asked to take off their shoes and stand with their back to the portable height meter/stadiometer. The sliding bar will be lowered and pressed flat on the head and recorded to the nearest centimeter. Body weight will be measured using a digital scale and weight will be recorded to the nearest gram. Age -and sex -specific prevalence of overweight and obesity will be determined by the body-mass- index for age Z scores using the criteria defined by the World Health Organisation (WHO). According to the WHO cut off points; overweight is considered to be > + 1SD (equivalent to BMI 25 kg/m2 at 19 years), Obesity: > + 2SD (equivalent to BMI 30 kg/m2 at 19 years), Thinness: <-2SD, Severe thinness: < −3SD and Normal: > −1 to < +1 SD. Standard deviations (Z-scores) will be computed using the World Health Organisation (WHO) reference population, 2007 soft ware (Anthropometric Software Program, Version 1.0.4 [41]. Blood pressure will be measured and the mean of three measurements will be recorded. Referrals will be initiated for children whose blood pressure exceeds the 95th percentile for age. Waist circumference will be measured to the nearest 0.1 cm horizontally at the narrowest point between the lower end of the rib cage and iliac crest. Hip circumference will be measured to the nearest 0.1 cm, at the greatest horizontal circumference below the iliac crest at the level of greater trochanter. Waist hip ratio will be obtained as waist circumference/hip circumference.
  The fitness level of the adolescents will be measured using the 20-m shuttle run test (20-mSRT) also known as the multi-stage fitness test. The 20-mSRT is the most widely used field-based test for aerobic fitness in young people and UK percentile data and health-related cut points are available [42]. A systematic review recently concluded that the 20-mSRT is the most reliable and valid field based method to estimate aerobic fitness in young people [43]. Performance in the 20-mSRT is typically expressed as laps, levels or distance completed. The 20mSRT estimates the cardiorespiratory fitness (aerobic capacity). Children run back and forth between two lines 20-m apart following beep signals played from a pre-recorded CD. The pace of the test is ramped such that running speed increases by 0.5 km/h each minute or period. The test is finished when the child stops owing to fatigue or when he/she does not reach the line in time with the audio signal on two consecutive occasions [44]. The last completed run indicates the final result of the test. The physical fitness data will be analysed and used as continuous variables and an increase in test results will indicate higher physical fitness [45].
(vi) Step count will be recorded using a pedometer. During the data collection process, 100 students (10% of the sample) will be asked to wear a Yamax SW200 Step Digi-Walker pedometer on their right hand for seven consecutive days, except during sleep and water based activities. Text messages/phone calls will be made daily as reminders to ensure compliance. The pedometers will be sealed with a sticker or cable to prevent reactivity i.e. a situation where individual change their normal activity pattern as a result of being monitored.
  The pedometers will be worn daily for seven days with a minimum of 4 valid days. Step counts of less than 1000 steps per day and greater than 30,000 steps per day will be treated as missing data [46]. Step count of 10,000 to 11,700 and over will be recorded as achieving the daily recommended levels of 60 min of MVPA in adolescents [47].

**Table 1:** Data Elements to be collected, Study Timepoints (T0, T1, T_2_), Target Groups

Objectives		Measure	Study instruments/Methods	Target group	Schedule		
					T_0_	T_1_	T_2_
Assess the pattern of physical activity among in-school adolescents		• PA Behaviours • Fitness	• PAQ-Questionnaire • Pedometers • 20-mSRT	Students	x	x	X
Identify factors (individual, social-cultural, built environment and policy) influencing physical activity of in-school adolescents	Individual	• Knowledge on PA, attitude, subjective norms (*normative beliefs and motivation to comply*) perceived behavioural control (*control beliefs and influence of control beliefs*), behavioural intentions and other demographic variables • Weight, Height, Waist-Hip circumference • 20-mSRT	• Questionnaire Focus group discussion guide • Anthropometric measurements • 20-mSRT	Students	x	x	x
	Socio -cultural environment	Social Norms Social support	• Key Informant Interview guide • Semi structured • Questionnaire • Focus Group Discussion guide	(i)Principals (ii)Officials of Ministries of Education, Health, Sports Physical and Health Education Teacher Classroom Teachers	x	x	x
	School’s built Environment	school grounds and safety, physical activity and recreation facilities aesthetics	• Observation using the SPEEDY Checklist	School Environment	x		x
	Policy	• PA Policies/programme • Extent of integration of recommendations in global and national policies and guidelines on physical activity for NCD prevention into the Nigerian school health policy	• Key Informant Interviews • Data extraction tool for Desk Review and Policy Analysis	(i) Principals (ii) PHE Teachers (iii) Officials of Ministries of Education, Health, Sports	x		x
Assess the effect of a multi-level intervention on the physical activity levels of secondary school students		• Knowledge on PA, attitude, subjective norms (*normative beliefs and motivation to comply*) perceived behavioural control (*control beliefs and influence of control beliefs*), behavioural intentions and other demographic variables • Weight, Height, Hip and Waist circumference • Fitness level	• Questionnaires • Pedometers • Anthropometric measurements • 20-mSRT	Students	x	x	x

T0: Baseline measurement

T1: Measurement immediately after the intervention

T2: Measurement three months after the intervention

### Primary outcome measurements

The primary outcome measures will be documented at baseline and final evaluation/follow-up. This include

(i) Self-reported physical activity levels among the students using the International Physical Activity Questionnaire for Adolescents
(ii) Step count recorded using a pedometer

### Secondary outcome measures

Secondary measures which will be documented are fitness levels, anthropometric and physical measurements, knowledge of the *health benefits of physical activity, attitude, self efficacy, perceived behavioural control and intention, Social Norms, Social support, policy or programme changes* on PA. Details in Table 2.

**Table 2:** Data elements to be collected, study timepoints (T0, T1, T2) target groups

Description of outcome measures	Method	Type of variable	Timing	Target group
Demographic characteristics, Socio-economic status	Questionnaire	Ordered categorical	T_0_ T_1_ T_2_	Adolescents
Knowledge, attitude, beliefs on nutrition and PA	Questionnaire	Ordered categorical	T_0_ T_1_ T_2_	Adolescents
Self-reported physical activity levels	Physical Activity Questionnaire (PAQ –A) for adolescents	Continuos	T_0_ T_1_ T_2_	Adolescents
		Ordered categorical		
PA Self efficacy	Questionnaire	Ordered categorical	T_0_ T_1_ T_2_	Adolescents
Perceived behavioural control and intention	Questionnaire	Ordered categorical	T_0_ T_1_ T_2_	Adolescents
Fitness level measured using the 20-mSRT	20-mSRT	Ordered categorical	T_0_ T_1_ T_2_	Adolescents
Step count (average steps/day)	Pedometers	Continuous	T_0_ T_1_ T_2_	Adolescents
Anthropometric and Physical assessments (BP, Ht, Wgt, BMI)	Brief physical assessment	Continuous	T_0_ T_1_ T_2_	Adolescents
Social Norms and Social support,	Questionnaire	Ordered categorical	T_0_ T_1_ T_2_	Adolescents, Teachers, School Heads
Built environment	Observation and checklist	Ordered categorical	T_0_	School Environment
Policy or programme changes	Key Informant interviews	Qualitative	T_0_ T_1_ T_2_	School Heads, Officials of Govt. and Ministries Policy Makers,

#### Intervention phase

The findings of the research phase will guide the development of the multi-level intervention targeting the policy makers, officials of the Ministries of Education, Health, Youths and Sports, School Heads and Physical and Health Education Teachers and the students. A *multilevel intervention is defined as an intervention* that addresses at least three levels of the multilayer system. It target at least three sources of influence that may ultimately result in improved health behaviour and outcomes [25]. Suggested activities which were identified based on literature [24, 48, 49] are outlined in Table 3.

**Table 3:** A list of multi-level Interventions based on a review of literature

Level	Possible Activities/Interventions
Individual Level	• Educational materials for students • Special Dance events in schools • Debates and other co-curricula activities • Experience sharing by role models/athletes • M health –SMS • Peer Education
Socio Cultural level	• Dialogue and dissemination meetings with Parent Teachers Association, Teachers, Principals
Built Environment	• Provision/Refurbishment of PA facilities
Policy/Institutional Level	• Development of curriculum &Training of Teachers • Review of PE Curriculum • Creating and training a school-based action team or small groups or committees. • Dissemination Meetings, • Production of evidence based dissemination materials – policy briefs, fact sheets etc. • One – on -one Advocacy visits • Celebration of special days and events

During baseline assessment, the students will be asked to rank a list of preferred activities which can be implemented in the school settings to improve physical activity. Activities which are under consideration include educational materials for students, special dance events in schools, debates and other co-curricula activities, experience sharing by role models/athletes, Mhealth and peer education. The preferred interventions will be jointly developed by the adolescents, school authorities and the researchers. At the school level, activities under consideration include meetings and policy dialogue forums, production of evidence based dissemination materials on the importance of physical activity for the health and mental well being of students, and advocacy visits to engage policymakers to prioritise resources aimed at promoting physical activity in schools. The policy goal for this intervention is to ensure that all in-school adolescents participate in 30 minutes of daily structured moderate to vigorous physical activity. Meetings will be held with Physical and Health Education Teachers to translate this policy objective into concrete activities and a work plan which will guide the intervention will be jointly developed. Issues which will be discussed at the meetings with the policy makers, physical health education teachers and principals include the perceived health implication of physical inactivity, risks of overweight and obesity among young people, ways to promote physical activity using existing infrastructure, allocation of 50% of physical education classes for activities. At the school level, a coordination committee will be set up to facilitate the implementation of the activities. The intervention phase will span three months and a post-intervention evaluation will be conducted to assess the outcome of this intervention.

The intervention will be implemented school wide however, the baseline and post-intervention measurements will only be conducted with the selected respondents enrolled as the cohort or index children at the commencement of the study.

### Monitoring of intervention

The intervention activities will be monitored through meetings with the school heads and Physical and Health Education Teachers to ascertain the extent of implementation of the activities.

### Validity

To ensure the validity of the tools, several steps will be taken. The tools will be adapted using simple English Language and if necessary, it will be translated into Yoruba Language to suit the Nigerian context. The tools will be reviewed for content and construct validity by specialists in physical activity and school health and pretested among similar respondents in selected schools in another LGA not used for the study.

### Reliability

To ascertain the reliability of the instrument, analysis of pre-test data will be done using Cronbach’s Alpha correlation coefficient of the IBM Statistical Package for Social Sciences (SPSS). A correlation coefficient greater than 0.7 will imply that the tool is reliable.

In order to ensure accurate and consistent measurements, the weighting scale will be calibrated with a known weight and the height scale will be checked and recalibrated daily before measurements commence.

Research assistants will be recruited and trained to assist in data collection and provision of support to students in completing the recall sections of the tools. Training sessions will include objectives of the study, importance of collecting accurate data and ethical issues. The questionnaire will be reviewed with them to ensure common understanding. They will be trained to relate skillfully and courteously with the respondents. At the end of the training session, the research assistants will role-play the administration of the questionnaires to demonstrate an understanding of the intricacies involved in data collection.

#### Data management and statistical analysis

Serial numbers will be assigned to the questionnaires for easy identification and recall of the instrument and these will be stored in a place safe. A coding guide will be developed and sections of the tools which are open ended will be coded before data entry. The data will be cleaned to identify and correct all errors. The quantitative data will be analyzed using STATA version 13.

There are two key primary outcome variables specifically step count measured with pedometers which is a continuous outcome variable and self-reported physical activity level which can be a continuos or ordered categorical variable, details in Table 4.

**Table 4:** Description of outcome Measures

	Description of outcome measures	Type of Variable
Primary Outcome Measures	Self-reported physical activity score/levels	Continuous
		Ordered categorical
	Step count (average steps/day)	Continuous
Secondary Outcome Measures	Anthropometric Measurements (measured using body mass index)	Ordered categorical
	Fitness level measured using the 20-mSRT	Ordered categorical
	Knowledge of the health benefits of physical activity	Ordered categorical
	Attitude toward Physical activity	Ordered categorical
	Self efficacy	Ordered categorical
	Perceived behavioural control and intention	Ordered categorical
	Social Norms and Social support,	Ordered categorical
	Policy or programme changes	Qualitative

At baseline, bivariate analysis (cross tabulation) will be used to compare the differences in proportions of the primary and secondary outcome measures of the experimental and control groups. Chi-square test (X^2^) will be used for significance testing of the self-reported physical activity levels (dependent ordered categorical variable) and other categorical independent variables while repeated measures ANOVA will be used for Step count (average steps/day). Based on data generated using bivariate analysis, all independent variables which are significant at 10% will be incorporated into the regression model.

Regression analysis will be used to assess the extent to which the independent variables predict self-reported physical activity levels and step count (average steps/day). Level of significance will be 5% at 95% confidence interval. The effect of the intervention will be tested using multilevel mixed effects models for repeated measures adjusted for relevant confounding factors. This approach ensures that the longitudinal and hierarchical structure of the data are considered in the analysis. Because of the clustered structure of the data, random effects for the cities and schools will be included in all analyses.

The qualitative data will be transcribed, coded, entered into NVIVO version 10 and analyzed using thematic approach. Points of agreement and disagreement among discussants with reference to the study objectives will be noted.

## Conclusion

This protocol outlines the rationale and description of a multi-level, multi-component, cluster randomised controlled trial study designed to improve the physical activity behaviours of in-school adolescents in south western Nigeria. The study is underpinned by the socio-ecological model and participatory research principles which involve study beneficiaries (adolescents) in the design of the intervention taking into cognizance the extraneous policy and environmental factors which influences their behaviours.

## Abbreviations

CONSORT: Consolidated Standards of Reporting Trials
ICC: Intraclass correlation coefficient
LGAs: Local government areas
MVPA: Moderate- to vigorous-intensity physical activity
NCDs: Non-communicable diseases
PA: Physical activity
PAQ-A: Physical Activity Questionnaire for Adolescents
RCT: Randomised controlled trial
S-PAPA: School Physical Activity Policy Assessment tool
SPEEDY: Sport, Physical activity and Eating behaviour: Environmental Determinants in Young people
TRA: Theory of Reasoned Action
