# Correction to: Effects of a multi-level intervention on the pattern of physical activity among in-school adolescents in Oyo state Nigeria: a cluster randomised trial

**DOI:** 10.1186/s12889-017-4885-4

**Published:** 2017-11-06

**Authors:** Mojisola Morenike Oluwasanu, Oladimeji Oladepo

**Affiliations:** African Regional Health Education Centre, Department of Health Promotion and Education, Faculty of Public Health, College of Medicine, University of Ibadan, Ibadan, Nigeria

## Correction

After publication of the article [1], it has been brought to our attention that there is an error in figure 1. The number of excluded secondary schools should read “50” and not “72”. The rest of the data in the figure is accurate.

## References

[1] Oluwasanu M, Oladepo O (2017). Effects of a multi-level intervention on the pattern of physical activity among in-school adolescents in Oyo state Nigeria: a cluster randomised trial. BMC Public Health.

